# 1,4-Bis(5-methyl-1*H*-1,2,4-triazol-3-yl)benzene tetra­hydrate

**DOI:** 10.1107/S1600536811015133

**Published:** 2011-04-29

**Authors:** Ai-Xin Zhu, Xiu-Li Chen, Zhen Li, Yuan-Chao Du, Hong-Can Wang

**Affiliations:** a Faculty of Chemistry and Chemical Engineering, Yunnan Normal University, Kunming 650092, People’s Republic of China; b Department of Chemistry, Zhengzhou Normal University, Zhengzhou 450044, People’s Republic of China

## Abstract

In the title compound, C_12_H_12_N_6_·4H_2_O, the two triazole rings adopt a *cis* configuration with a crystallographic twofold axis passing through the central benzene group. The benzene and triazole rings are almost coplanar with a dihedral angle of 5.5 (1)°. In the crystal, water mol­ecules are joined together by O*W*—H⋯O*W* hydrogen bonds to form a one-dimensional zigzag chain. These water chains are further connected to the organic mol­ecule, forming a three-dimensional network by inter­molecular O*W*—H⋯N and N—H⋯O*W* hydrogen bonds. Moreover, π–π stacking inter­actions between triazole rings [centroid–centroid distances = 3.667 (1)–3.731 (1) Å] are observed. One of the water mol­ecules shows one of the H atoms to be disordered over two positions.

## Related literature

For applications of 1,2,4-triazole and its derivatives in coordination chemistry, see: Zhang *et al.* (2005 ▶); Ouellette *et al.* (2006 ▶); Zhu *et al.* (2009 ▶). For the structures of ruthenium complexes with pyridine-2-yl-1,2,4-triazole-based ligands, see: Passaniti *et al.* (2002 ▶). For the previous synthesis of the title compound, see: Bahçeci *et al.* (2005 ▶).
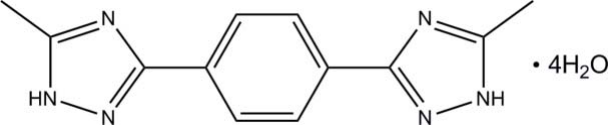


## Experimental

### 

#### Crystal data

C_12_H_12_N_6_·4H_2_O
*M* *_r_* = 312.34
Monoclinic, 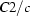

*a* = 12.7343 (19) Å
*b* = 13.937 (2) Å
*c* = 9.0648 (14) Å
β = 100.893 (3)°
*V* = 1579.8 (4) Å^3^
*Z* = 4
Mo *K*α radiation
μ = 0.10 mm^−1^
*T* = 293 K
0.35 × 0.28 × 0.08 mm

#### Data collection

Bruker APEX CCD diffractometer
Absorption correction: multi-scan (*SADABS*; Sheldrick, 1996 ▶) *T*_min_ = 0.966, *T*_max_ = 0.992
4670 measured reflections
1542 independent reflections
1286 reflections with *I* > 2σ(*I*)
*R*_int_ = 0.021

#### Refinement

*R*[*F* ^2^ > 2σ(*F* ^2^)] = 0.052
*wR*(*F* ^2^) = 0.148
*S* = 1.04
1542 reflections
102 parameters
H-atom parameters constrained
Δρ_max_ = 0.27 e Å^−3^
Δρ_min_ = −0.25 e Å^−3^

### 

Data collection: *SMART* (Bruker, 2004 ▶); cell refinement: *SAINT* (Bruker, 2004 ▶); data reduction: *SAINT*; program(s) used to solve structure: *SHELXS97* (Sheldrick, 2008 ▶); program(s) used to refine structure: *SHELXL97* (Sheldrick, 2008 ▶); molecular graphics: *DIAMOND* (Brandenburg, 1999 ▶); software used to prepare material for publication: *SHELXTL* (Sheldrick, 2008 ▶).

## Supplementary Material

Crystal structure: contains datablocks I, global. DOI: 10.1107/S1600536811015133/im2280sup1.cif

Supplementary material file. DOI: 10.1107/S1600536811015133/im2280Isup2.cdx

Structure factors: contains datablocks I. DOI: 10.1107/S1600536811015133/im2280Isup3.hkl

Supplementary material file. DOI: 10.1107/S1600536811015133/im2280Isup4.cml

Additional supplementary materials:  crystallographic information; 3D view; checkCIF report

## Figures and Tables

**Table 1:** Hydrogen-bond geometry (Å, °)

*D*—H⋯*A*	*D*—H	H⋯*A*	*D*⋯*A*	*D*—H⋯*A*
N1—H1*D*⋯O1*W*	0.86	1.88	2.736 (2)	173
O1*W*—H1*WA*⋯N2^i^	0.85	2.08	2.926 (2)	172
O1*W*—H1*WB*⋯O2*W*^ii^	0.85	1.96	2.801 (2)	170
O2*W*—H2*WA*⋯N3^iii^	0.85	1.95	2.800 (2)	173
O2*W*—H2*WB*⋯O2*W*^iii^	0.85	1.93	2.754 (3)	164
O2*W*—H2*WC*⋯O2*W*^iv^	0.85	1.92	2.774 (3)	178

Symmetry codes: (i) 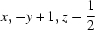; (ii) 
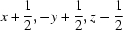
; (iii) 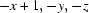; (iv) 
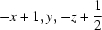
.

**Fig. 1.**
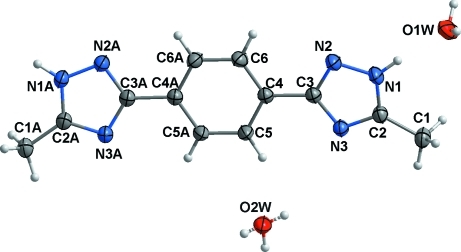
Molecular structure of the title compound, non-H atoms are depicted as 30% probability displacement ellipsoids.

**Fig. 2.**
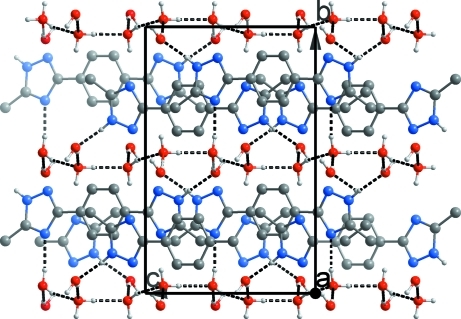
Packing diagram of the title compound showing the hydrogen bonding interactions as dashed lines. H atoms not involved in hydrogen bondings have been omitted.

**Fig. 3.**
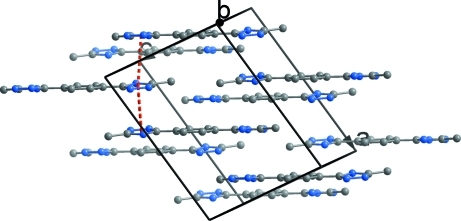
π-π Stacking interactions between triazole rings, H and O atoms are omitted for clarity.

## supplementary crystallographic information

### Comment

In the past few years, 1,2,4-triazole and its derivatives have attracted increasing attention as *N*-heterocyclic aromatic ligands, since they are effective bridging ligands combining the coordination modes of both imidazoles and pyrazoles. In addition, metal-triazolate frameworks have demonstrated high thermal and chemical stabilities, and interesting luminescent, magnetic and gas-adsorption properties (Zhang *et al.* 2005; Ouellette *et al.* 2006; Zhu *et al.* 2009). However, there are rare crystal structure reports of 1,2,4-triazole derivatives attached to aromatic groups (Passaniti *et al.* 2002). Although the synthesis of the title compound 1,4-bis(5-methyl-1*H*-1,2,4-triazol-3-yl)benzene has been reported by Bahçeci *et al.* (2005), no crystallographic study has been reported on this ligand and related metal coordination compounds. We reported herein another synthetic method and the crystal structure of the title compound.

The asymmetric unit of the title compound contains one-half organic molecule, which adopts a *cis*-configuration with a crystallographic mirror plane passing through the central benzene group, and two water molecules (Fig. 1). The bond lengths and angles are within normal ranges in accordance with the corresponding values reported (Passaniti *et al.* 2002). The benzene and the triazole rings are almost coplanar, with a dihedral angle of 5.4 (1)°. In the crystal structure, water molecules are joined together by OW—H···OW hydrogen bonds to form a one-dimensional zig-zag water chain (Fig. 2, Table 1). These water chains are further connected to the organic molecule producing a three-dimensional network (Fig. 2) by intermolecular OW—H···N and N—H···OW hydrogen bonds (Table 1). Moreover, π-π stacking interactions between triazole rings (centroid-centroid distance = 3.665 (1)–3.732 (1) Å) are observed (Fig. 3).

### Experimental

The ligand 1,4-bis(5-methyl-1*H*-1,2,4-triazol-3-yl)benzene was synthesized according to a literature method (Bahçeci *et al.* 2005). Yellow, plate-like single crystals of the title compound are obtained from a solution of 1,4-bis(5-methyl-1*H*-1,2,4-triazol-3-yl)benzene (24 mg, 0.1 mmol) in methanol (1 ml) and water (5 ml) if the solution is placed in a Teflon-lined stainless steel vessel (15 ml), heated at 453 K for 24 h and then cooled to room temperature at a rate of 5 K h^-1^.

### Refinement

All H atoms were placed in idealized positions (O—H = 0.85 Å, N—H = 0.86 Å and C—H = 0.95 Å) and refined as riding atoms with *U*_iso_(H) = 1.2*U*_eq_(C, N) and *U*_iso_(H) = 1.5*U*_eq_(O). One hydrogen atom from O2W is disordered over two positions in a 0.52 (3):0.48 (3) ratio, which is freely refined with the command 'PART'.

### Crystal data

**Table:**

C_12_H_12_N_6_·4H_2_O	*Z* = 4
*M**_r_* = 312.34	*F*(000) = 664
Monoclinic, *C*2/*c*	*D*_x_ = 1.313 Mg m^−^^3^
Hall symbol: -C 2yc	Mo *K*α radiation, λ = 0.71073 Å
*a* = 12.7343 (19) Å	µ = 0.10 mm^−^^1^
*b* = 13.937 (2) Å	*T* = 293 K
*c* = 9.0648 (14) Å	Plate, yellow
β = 100.893 (3)°	0.35 × 0.28 × 0.08 mm
*V* = 1579.8 (4) Å^3^	

### Data collection

**Table:**

Bruker APEX CCD diffractometer	1542 independent reflections
Radiation source: fine-focus sealed tube	1286 reflections with *I* > 2σ(*I*)
graphite	*R*_int_ = 0.021
ω scans	θ_max_ = 26.0°, θ_min_ = 2.2°
Absorption correction: multi-scan (*SADABS*; Sheldrick, 1996)	*h* = −15→15
*T*_min_ = 0.966, *T*_max_ = 0.992	*k* = −15→17
4670 measured reflections	*l* = −11→11

### Refinement

**Table:**

Refinement on *F*^2^	Primary atom site location: structure-invariant direct methods
Least-squares matrix: full	Secondary atom site location: difference Fourier map
*R*[*F*^2^ > 2σ(*F*^2^)] = 0.052	Hydrogen site location: inferred from neighbouring sites
*wR*(*F*^2^) = 0.148	H-atom parameters constrained
*S* = 1.04	*w* = 1/[σ^2^(*F*_o_^2^) + (0.0816*P*)^2^ + 0.7602*P*] where *P* = (*F*_o_^2^ + 2*F*_c_^2^)/3
1542 reflections	(Δ/σ)_max_ < 0.001
102 parameters	Δρ_max_ = 0.27 e Å^−^^3^
0 restraints	Δρ_min_ = −0.25 e Å^−^^3^

### Special details

**Table:**

Geometry. All e.s.d.'s (except the e.s.d. in the dihedral angle between two l.s. planes) are estimated using the full covariance matrix. The cell e.s.d.'s are taken into account individually in the estimation of e.s.d.'s in distances, angles and torsion angles; correlations between e.s.d.'s in cell parameters are only used when they are defined by crystal symmetry. An approximate (isotropic) treatment of cell e.s.d.'s is used for estimating e.s.d.'s involving l.s. planes.
Refinement. Refinement of *F*^2^ against ALL reflections. The weighted *R*-factor *wR* and goodness of fit *S* are based on *F*^2^, conventional *R*-factors *R* are based on *F*, with *F* set to zero for negative *F*^2^. The threshold expression of *F*^2^ > σ(*F*^2^) is used only for calculating *R*-factors(gt) *etc*. and is not relevant to the choice of reflections for refinement. *R*-factors based on *F*^2^ are statistically about twice as large as those based on *F*, and *R*- factors based on ALL data will be even larger. One hydrogen atom from O2W is disordered over two positions in a 0.52 (3):0.48 (3) ratio, which is freely refined with command 'PART'.

### Fractional atomic coordinates and isotropic or equivalent isotropic displacement parameters (Å^2^)

**Table:**

	*x*	*y*	*z*	*U*_iso_*/*U*_eq_	Occ. (<1)
N1	0.66106 (13)	0.34969 (11)	−0.19565 (17)	0.0531 (5)	
H1D	0.6874	0.3847	−0.2578	0.064*	
N2	0.62354 (14)	0.38395 (11)	−0.07499 (17)	0.0522 (5)	
N3	0.60877 (12)	0.22386 (10)	−0.09140 (16)	0.0454 (4)	
C1	0.68428 (18)	0.19667 (15)	−0.3248 (2)	0.0590 (6)	
H1A	0.7292	0.1449	−0.2805	0.088*	
H1B	0.7230	0.2361	−0.3830	0.088*	
H1C	0.6218	0.1711	−0.3887	0.088*	
C2	0.65166 (14)	0.25529 (13)	−0.20444 (18)	0.0448 (4)	
C3	0.59264 (14)	0.30486 (12)	−0.01538 (19)	0.0425 (4)	
C4	0.54472 (14)	0.30544 (12)	0.12072 (19)	0.0421 (4)	
C5	0.52248 (17)	0.22014 (13)	0.1863 (2)	0.0510 (5)	
H5A	0.5379	0.1622	0.1443	0.061*	
C6	0.52170 (16)	0.39108 (13)	0.1860 (2)	0.0510 (5)	
H6A	0.5358	0.4491	0.1429	0.061*	
O1W	0.73472 (14)	0.45314 (11)	−0.41166 (19)	0.0757 (5)	
H1WA	0.6969	0.4969	−0.4609	0.091*	
H1WB	0.8001	0.4691	−0.4017	0.091*	
O2W	0.44076 (12)	−0.02791 (9)	0.10493 (16)	0.0602 (4)	
H2WA	0.4240	−0.0868	0.0928	0.072*	
H2WB	0.4790	−0.0006	0.0500	0.072*	0.52 (3)
H2WC	0.4784	−0.0277	0.1930	0.072*	0.48 (3)

### Atomic displacement parameters (Å^2^)

**Table:**

	*U*^11^	*U*^22^	*U*^33^	*U*^12^	*U*^13^	*U*^23^
N1	0.0657 (11)	0.0516 (10)	0.0484 (8)	−0.0032 (8)	0.0274 (8)	0.0074 (7)
N2	0.0662 (11)	0.0437 (9)	0.0523 (9)	−0.0025 (7)	0.0257 (8)	0.0035 (7)
N3	0.0540 (9)	0.0428 (8)	0.0425 (8)	−0.0035 (7)	0.0166 (7)	−0.0015 (6)
C1	0.0649 (13)	0.0672 (13)	0.0495 (10)	−0.0045 (10)	0.0232 (9)	−0.0060 (9)
C2	0.0467 (10)	0.0488 (10)	0.0401 (9)	−0.0022 (8)	0.0115 (7)	0.0016 (7)
C3	0.0452 (10)	0.0437 (9)	0.0397 (9)	−0.0003 (7)	0.0108 (7)	0.0012 (7)
C4	0.0448 (10)	0.0431 (10)	0.0396 (9)	−0.0001 (7)	0.0109 (7)	0.0010 (7)
C5	0.0774 (14)	0.0368 (9)	0.0429 (9)	0.0004 (9)	0.0218 (9)	−0.0027 (7)
C6	0.0603 (12)	0.0380 (10)	0.0604 (11)	−0.0004 (8)	0.0260 (9)	0.0049 (8)
O1W	0.0804 (11)	0.0679 (10)	0.0871 (11)	0.0058 (8)	0.0373 (9)	0.0277 (8)
O2W	0.0809 (11)	0.0447 (7)	0.0611 (9)	−0.0064 (7)	0.0289 (8)	−0.0014 (6)

### Geometric parameters (Å, °)

**Table:**

N1—C2	1.322 (3)	C4—C5	1.382 (2)
N1—N2	1.360 (2)	C4—C6	1.388 (2)
N1—H1D	0.8600	C5—C5^i^	1.383 (4)
N2—C3	1.320 (2)	C5—H5A	0.9300
N3—C2	1.324 (2)	C6—C6^i^	1.376 (4)
N3—C3	1.358 (2)	C6—H6A	0.9300
C1—C2	1.484 (3)	O1W—H1WA	0.8500
C1—H1A	0.9600	O1W—H1WB	0.8500
C1—H1B	0.9600	O2W—H2WA	0.8500
C1—H1C	0.9600	O2W—H2WB	0.8501
C3—C4	1.476 (2)	O2W—H2WC	0.8499
			
C2—N1—N2	110.87 (14)	N2—C3—C4	122.67 (15)
C2—N1—H1D	124.6	N3—C3—C4	123.69 (15)
N2—N1—H1D	124.6	C5—C4—C6	118.63 (17)
C3—N2—N1	102.31 (15)	C5—C4—C3	120.35 (15)
C2—N3—C3	104.00 (15)	C6—C4—C3	121.01 (15)
C2—C1—H1A	109.5	C4—C5—C5^i^	120.67 (10)
C2—C1—H1B	109.5	C4—C5—H5A	119.7
H1A—C1—H1B	109.5	C5^i^—C5—H5A	119.7
C2—C1—H1C	109.5	C6^i^—C6—C4	120.69 (10)
H1A—C1—H1C	109.5	C6^i^—C6—H6A	119.7
H1B—C1—H1C	109.5	C4—C6—H6A	119.7
N1—C2—N3	109.18 (15)	H1WA—O1W—H1WB	108.3
N1—C2—C1	123.88 (16)	H2WA—O2W—H2WB	121.0
N3—C2—C1	126.93 (17)	H2WA—O2W—H2WC	102.0
N2—C3—N3	113.64 (16)	H2WB—O2W—H2WC	105.4

Symmetry codes: (i) −*x*+1, *y*, −*z*+1/2.

### Hydrogen-bond geometry (Å, °)

**Table:**

*D*—H···*A*	*D*—H	H···*A*	*D*···*A*	*D*—H···*A*
N1—H1D···O1W	0.86	1.88	2.736 (2)	173.
O1W—H1WA···N2^ii^	0.85	2.08	2.926 (2)	172.
O1W—H1WB···O2W^iii^	0.85	1.96	2.801 (2)	170.
O2W—H2WA···N3^iv^	0.85	1.95	2.800 (2)	173.
O2W—H2WB···O2W^iv^	0.85	1.93	2.754 (3)	164.
O2W—H2WC···O2W^i^	0.85	1.92	2.774 (3)	178.

Symmetry codes: (ii) *x*, −*y*+1, *z*−1/2; (iii) *x*+1/2, −*y*+1/2, *z*−1/2; (iv) −*x*+1, −*y*, −*z*; (i) −*x*+1, *y*, −*z*+1/2.
